# Narrative Review of the Post-Operative Management of Prostate Cancer Patients: Is It Really the End of Adjuvant Radiotherapy?

**DOI:** 10.3390/cancers14030719

**Published:** 2022-01-30

**Authors:** Vincent Bourbonne, Olivier Pradier, Ulrike Schick

**Affiliations:** Radiation Oncology Department, University Hospital, 29200 Brest, France; olivier.pradier@chu-brest.fr (O.P.); ulrike.schick@chu-brest.fr (U.S.)

**Keywords:** adjuvant radiotherapy, early salvage radiotherapy, personalized medicine, biomarker

## Abstract

**Simple Summary:**

Among patients with prostate cancer who have been operated on, a subset harboring high-risk features will present with a biochemical recurrence (BCR). Adjuvant radiotherapy (aRT) was proven to significantly reduce the risk of BCR when compared to salvage radiotherapy (SRT) but suffered from several limitations: a lack of patient selection criteria, a higher treatment-related morbidity and an uncertain benefit for long-term clinical endpoints. In the same clinical setting, early SRT (eSRT) appears as non-inferior to aRT with a lower morbidity, replacing aRT as the preferred option. In this review, we insist on the need for multidisciplinary discussions to fully comprehend the individual characteristics of each patient and propose the best treatment strategy for every patient.

**Abstract:**

Despite three randomized trials indicating a significant reduction in biochemical recurrence (BCR) in high-risk patients, adjuvant radiotherapy (aRT) was rarely performed, even in patients harboring high-risk features. aRT is associated with a higher risk of urinary incontinence and is often criticized for the lack of patient selection criteria. With a BCR rate reaching 30–70% in high-risk patients, a consensus between urologists and radiation oncologists was needed, leading to three different randomized trials challenging aRT with early salvage radiotherapy (eSRT). In these three different randomized trials with event-free survival as the primary outcome and a planned meta-analysis, eSRT appeared as non-inferior to aRT, answering, for some, this never-ending question. For many, however, the debate persists; these results raised several questions among urologists and radiation oncologists. BCR is thought to be a surrogate for clinically meaningful endpoints such as overall survival and cancer-specific survival but may be poorly efficient in comparison with metastasis-free survival. Imaging of rising prostate-specific antigen (PSA), post-operative persistent PSA and BCR was revolutionized by the broader use of MRI and nuclear imaging such as PET-PSMA; these imaging modalities were not analyzed in the previous randomized trials. A sub-group of very high-risk patients could possibly benefit from an adjuvant radiotherapy; but their usual risk factors such as high Gleason score or invaded surgical margins mean they are unable to be selected. More precise biomarkers of early BCR or even metastatic-relapse were developed in this setting and could be useful for the patients’ stratification. In this review, we insist on the need for multidisciplinary discussions to fully comprehend the individual characteristics of each patient and propose the best treatment strategy for every patient.

## 1. Introduction

Approximately 30% of operated on prostate cancer (PCa) patients will experience biochemical recurrence (BCR), this rising to 50–70% in very high-risk patients [1,2,3]. Since 2009–2012 and the publications of three randomized controlled trials [1,4,5] (RCTs) evaluating the benefit of adjuvant radiotherapy (aRT) in PCa patients with high-risk features, a gap has opened up between urologists/radiation oncologists who believed these three trials to be practice-changing and those who awaited confirmation of these results on more robust endpoints. Despite a significant benefit for biochemical-free recurrence (bRFS), the only trial in favor of a significant benefit on metastasis-free survival (MFS) and overall survival (OS) was criticized for its design [4]. These debates resulted in a low use of aRT, clinicians fearing the higher genitourinary morbidity associated with aRT when compared to salvage radiotherapy (SRT). The benefit on OS was difficult to evaluate in a population with an expected survival higher than the proposed follow-up.

Given the possible lack of benefit on OS and its higher toxicity profile, aRT was challenged by early SRT (eSRT) in three recently published RCTs [6,7,8] pooled in a pre-planned meta-analysis [9] that proved eSRT to be non-inferior to aRT when considering event-free survival (EFS harmoniously defined as the time from randomization until the first evidence of either biochemical progression (prostate-specific antigen (PSA) ≥ 0.4 ng/mL and rising after completion of any post-operative radiotherapy), clinical or radiological progression, initiation of a non-trial treatment, death from prostate cancer or a PSA level of at least 2.0 ng/mL at any time after randomization) with a toxicity profile in favor of eSRT over aRT. For some, these trials closed the discussion between aRT and (e)SRT. 

Several reserves must be raised before closing the debate so rapidly. The number of events was quite low in the three trials with a relatively short follow-up. Despite a non-significant benefit in the ARTISTIC meta-analysis, the EFS favored aRT over eSRT in the GETUG-AFU17 and the RAVES trials. Inclusion criteria and study design substantially differed from one trial to another, especially regarding the inclusion/exclusion of patients with a lymph node invasion (LNI). Contemporary imaging modalities such as PET-PSMA were not used, and the performances of recent prognostic tools such as genomic tests [10] were not used or evaluated.

In this review, we aim to revisit the place of aRT and eSRT in patients operated on with high-risk PCa and give an overview of the tools available for personalizing the best treatment selection for each patient.

## 2. Adjuvant Radiotherapy Versus Salvage Radiotherapy

The prostate-specific antigen (PSA) level at the time of post-operative RT was proved to be critical, with a high correlation between the PSA value and the risk of bRFS [11]. Pre-SRT PSA is often used in prediction models among other features such as the PSA doubling time, the use of concomitant or neoadjuvant androgen-deprivation therapy (ADT), the pathology Gleason grade, the surgical margins as well as the presence of LNI [11]. Performance of aRT versus SRT, but only in patients who presented a high-risk of BCR, was thus evaluated. Three RCTs were conducted with different inclusion/exclusion criteria. With a cohort of 425 men with a T3N0M0 PCa treated either with a 60 to 64 Gy aRT or observation, the SWOG S8794 trial showed that aRT reduces the risk of metastasis and benefits OS [4]. It is to be noted that a non-negative post-operative PSA was not considered as an exclusion criteria and 33.4% of the overall population had a post-operative PSA > 0.2 ng/mL. A similar rate of non-negative post-operative PSA was observed in the EORTC 22911 RCT (rate of 29.9% in the overall cohort) [1]. Only the ARO 96-02/AUO AP 09/95 study considered this situation as a progression and, thus, as an exclusion criterion [5]. Considering the inconsistency of the post-operative PSA level, some would argue that these trials are more a comparison between (early) SRT vs. delayed SRT than aRT vs. observation. Furthermore, the three RCTs had substantial differences regarding other inclusion criteria: only pT3N0 patients were included in the SWOG S8794 [4], while ARO 96-02/AUO AP 09/95 [5] included pT4 patients and patients in EORTC 22911 [1] had at least two risk features. 

Among the three RCTs, only the SWOG S8794 trial used metastasis recurrence-free survival as the primary endpoint, while the EORTC 22911 used bRFS and ARO 96-02/AUO AP 09/95 a progression-free survival (PFS), but defined as the non-occurrence of BCR, local or distant clinical recurrence or death from any cause. The three RCTs met their respective primary endpoints, but both EORTC 22911 and ARO 96-02/AUO AP 09/95 failed to prove the benefit of aRT on MFS and OS (secondary endpoints). Results from these three RCTs were pooled in a meta-analysis conducted by the Cochrane Library [12]. Despite the presented biases of these trials, this meta-analysis concluded to an improvement of OS and MFS with aRT over observation but only with a longer follow-up. To be reminded, the mean follow-up durations in the SWOG S8794, EORTC 22911 and ARO 96-02/AUO AP 09/95 trials were approximately 12.5, 10.6 and 9.4 years, respectively. A meta-analysis published several years before concluded to the absence of an OS benefit of aRT, supporting the absolute need of a longer follow-up [13].

Since these three RCTs, a fourth RCT has also been presented confirming the bRFS benefit of aRT vs. observation but with a mean follow-up of 8.6 years in the observation group, with no benefit on MFS and OS [14].

In spite of this level A data, aRT remained widely underused. For instance, in the United States of America, between 2004 and 2015, only 11.7% of the 189,240 eligible patients (adverse events on pathology reports) received aRT [15]. The rate rose to 28.9% in the very high-risk cohort (at least two risk features among ≥pT3b, pathological Gleason 8–10 and pN1). This under-utilization reflects the puzzlement of urologists and radiation oncologists regarding a clinical setting in which only 30–70% of the patients will present with an actual BCR and eventually benefit from aRT. The other 30–70% would experience a higher grade 2 genitourinary morbidity with no actual clinical benefit [1,4,5]. For completeness, the results of these three RCTs are available as Table 1.

Genitourinary morbidity after post-operative radiotherapy is correlated to the delay between surgery and start of RT and the patient’s complete functional recovery [16]. Two conflicting issues, thus, seem to oppose themselves: the EFS and the quality of life of our patients.

## 3. Adjuvant Radiotherapy Versus Early Salvage Radiotherapy

Early salvage radiotherapy (eSRT) is defined as the delivery of SRT to patients with low level rising PSA values (PSA > 0.1–0.2 ng/mL). Use of eSRT is supported by solid retrospective data such as multi-institutional retrospective studies [17]. With 510 included patients, no significant differences in terms of distant metastasis and mortality when compared to aRT were observed. This absence of significant difference in mortality and the lower rate of genitourinary morbidity when compared to aRT led to eSRT being often preferred by some urologists, even in patients harboring pathological risk factors. Three separate RCTs were thus conducted to evaluate the benefit of aRT over eSRT. Again, these RCTs differ on their respective primary endpoints and inclusion/exclusion criteria but were pooled in a planned meta-analysis [9]. None of the RADICALS-RT, RAVES or GETUG-17 demonstrated a significant benefit on MFS or OS (secondary endpoints), the respective endpoints being bRFS for the RAVES trial and EFS for the RADICALS-RT and GETUG-17 trials. Interestingly, the median follow-up was significantly shorter than the “aRT vs. observation” trials with respective FUs of 4.9, 6.1 and 6.3 years. The only positive RCT on aRT vs. observation for MFS was that with the longest follow-up (14.7 years for the aRT cohort and 12.9 for the observation cohort). Such a short follow-up is especially troublesome in this setting because of several points:

- When given (RADICALS-RT and GETUG-AFU-17), ADT artificially prolongs bRFS. Indeed, BCR under ADT at this stage of the disease almost never happens. On the aRT arm, ADT is administered shortly after the randomization and probably 1 to 2 months before the start of aRT. On the eSRT arm, ADT and eSRT could start concomitantly. This design adds a systematic bias that probably negatively impacts the aRT’s results
- Because of the definition of BCR, patients with the same biochemical control can be classified differently only because of their affected treatment group. For instance, in the GETUG-AFU-17 trial: BCR will be reached as soon as the PSA rises to 0.4 ng/mL at least 6 months after RT completion for the aRT arm, whereas in the SRT arm, BCR will be reached at the time of the follow-up meetings several months after RT completion. Similarly, defining the bRFS from the date of randomization contributes to this statistical bias. This difference adds a systematic extension of the bRFS in the SRT arm that is problematic with such a short follow-up. This directly induces a possible bias as observed in the RAVES trial with a 5-year BCR-free rate of 86% and 87% for the aRT and eSRT arm, while the 8-year BCR-free rate fell to 80% and 75% for the aRT and eSRT arms, respectively [7].
- The GETUG-AFU-17 trial was closed prematurely because of the low rate of events.

The results from the three RCTs (summarized in Table 2) were pooled in the ARTISTIC meta-analysis that seemed to confirm the “observation” attitude [9], with the authors concluding to the absence of benefit of aRT over eSRT for all patients. None of the patients seemed to significantly benefit from aRT, even on the sub-group analysis. It is to be noted that patients with a Gleason score ≥ 8, a high CAPRA-S risk group and especially patients with an LNI were insufficiently represented (respective rates of 15.1%, 35.2% and 3.9%). Data on very high-risk patients (CAPRA-S risk score ≥ 8) are unavailable. Knowing the 2.4-fold increase in risk with a 2-point increase on the CAPRA-S, these detailed data are needed [18]. These very high-risk patients are those who probably benefit the most from a post-operative treatment [19]. Unfortunately, in spite of their efforts, data regarding very high-risk patients are inconclusive and need further research. Among the risk features, LNI needs a specific focus given its low representation in the previously presented trials.

## 4. Impact of an LNI in the Choice of aRT and eSRT

LNI is known to be a major risk feature for PCa patients because it represents a shift in the disease from a localized to a metastatic state. Management of these patients is, thus, a priority. To our knowledge, no prospective trial evaluating the benefit of aRT over SRT or (e)SRT has focused specifically on patients with a pN1 status [20].

LNI was an exclusion criteria for the three RCTs comparing aRT and observation [1,4,5]. Retrospective data suggest a clinical benefit of aRT over ADT alone in certain subsets of patients. Adjuvant radiotherapy significantly impacts cancer-specific mortality in two sub-groups of patients: patients with 1 to 2 positives nodes with a Gleason score 7–10 or pT3b/PT4/positive margins (group 3) or patients with 3–4 positives nodes (group 4) [21]. Patients with ≥4 positive nodes probably were micro-metastatic. The rest of the patients did not harbor enough risk features to benefit from aRT as previously proven [19]. aRT was systematically associated with ADT. This simple selection tool was externally validated with a significant reduction in overall mortality in the aRT + ADT arm when compared to the ADT alone arm. In Groups 3 and 4, hazard ratios were respectively 0.75 (0.62–0.91) and 0.57 (0.38–0.86) [22]. Even without this group selection, the addition of aRT to ADT in pN1 patients still appears to be beneficial to overall survival [23].

As previously stated, only two of the three RCTs pooled in the ARTISTIC meta-analysis allowed patients with LNI to be included. Patients with LNI represent a very small subset of the overall cohort (3.0%). No conclusions regarding the impact of LNI on the effect of RT timing could thus be proposed, leaving clinicians wondering about the generalizability of the aRT vs. eSRT results to the pN1 patients.

## 5. Impact of ADT on the Choice of aRT and eSRT

A major possible confounding factor was the possibility (or non-possibility) of concomitant ADT, depending on the RCT. The addition of 24 months of ADT to SRT was significantly associated with longer MFS and OS in the RTOG 9601 trial [24]. The CAPRA-S score was not evaluable in this specific study but, again, a low rate of Gleason ≥ 8 score is observed (17.3%) and a low PSA level at the time of randomization, with 53.3% of the patients having a PSA level < 0.7 ng/mL. However, this benefit of ADT was not consistent across the whole range of patients. On a multicentric cohort, only patients with more aggressive characteristics at the time of SRT (pT3b/4 and grade group ≥ 4 or pT3b/4 and pre-SRT PSA ≥ 0.4 ng/mL) had a significantly better MFS with ADT [25]. The value of the pre-SRT PSA level was confirmed as an efficient surrogate for the benefit of ADT. In patients with a pre-SRT PSA level < 0.6 ng/mL, the benefit of adding ADT on OS was not significant, probably because of the lower OS benefit counterbalanced by the cardiovascular morbidity associated with ADT [26]. Pre-SRT PSA thus stands out as a potential prognostic biomarker for patient selection before ADT + SRT. Delivered in selected patients with a very high-risk profile but a long life expectancy and no post-operative dysfunction, aRT could be seen as an effective possibility to postpone ADT. Furthermore, data supporting the addition of ADT to aRT remain scarce, with several studies reporting the absence of an OS benefit [27], probably due to the low PSA value at the time of aRT.

A note must be made regarding the design of the six RCTs focusing on eSRT, SRT and aRT. Apart from the RADICALS trial, none of the RCTs were designed or powered enough to evaluate the impact of ADT in the post-operative setting.

## 6. Optimizing the Selection of Patients for aRT: Novel Biological and Diagnostic Approaches

All the data seem to converge towards a single challenge, namely the selection of patients for either aRT or (e)SRT. Numerous clinical features are taken into account, with a consensus being adopted on the definition of high-risk and very high-risk patients. Data on high-risk patients are temporary awaiting a longer follow-up, while data on very high-risk patients are scarce or unavailable. The ARTISTIC meta-analysis used the CAPRA-S risk score to combine risk features and stratify the included patients but did not find a significant benefit of aRT over eSRT, even in patients with a high CAPRA-S score. Being an aggregate study-level analysis and not an individual patient-level analysis, all subset analyses in the ARTISTIC meta-analysis were probably underpowered. Before detailing other selection modalities, the importance of a central pathology review must be stressed [28,29]. Pathology review is a major possible cofounding factor in these RCTs in which inclusion criteria are pathology-based. On the three RCTs evaluating aRT vs. eSRT, none had a central pathology review for all patients. Apart from clinical and pathology-based features, other selection modalities were developed. Firstly, one could think disease staging at the time of aRT/SRT could impact the treatment’s outcomes. For example, patients, with an unknown LNI status at the time of post-operative RT and who would be treated with SRT delivered to the prostate fossa only, would not benefit from SRT. With its high sensitivity, 68-GA-PSMA positron emission tomography (PET) appears as the most efficient imaging modality to detect an eventual LNI. However, despite its high sensitivity, performing such an exam did not significantly impact patients’ outcomes [30], probably because of the low rate of detection in the case of PSA < 0.2 ng/mL when aRT or eSRT is performed [31]. PSMA PET associated with a magnetic resonance imaging could enhance the detection rate [32] and possibly modify the definition of radiotherapy target volumes [33,34]. Several clinicians are currently rethinking their approach in the case of BCR, incorporating PSMA PET as a tool to guide diagnostic or therapeutic management. Differing treatments until PSMA PET reveals a relapse site amenable to targeted radiotherapy may be tempting, but given the prognostic value of a negative PSMA PET in the post-operative setting [35], differing RT should only be performed within clinical trials.

Characterization of the disease with genomic or radiomics approaches also appear as an efficient tools for patients’ selection. In this setting and on a sub-analysis of 486 patients, a genomic-classifier was significantly correlated with MFS and OS [10]. The Decipher test impacts decision making among patients considered for aRT/eSRT and is an independent predictor of response to RT [36,37]. The Decipher tests stratifies patients among three risk-based cohorts. Patients with a score > 0.6 have the highest risk of recurrence [38]. Such a tool could guide the selection of patients for ADT, aRT/SRT or observation, with a cut-off of 0.6 [10]. A radiomics model based on a sole radiomic feature was externally validated as an efficient tool for the stratification of patients based on their BCR risk [39]. Combining these genomics and radiomics approaches (“radiogenomics”) resulted in higher performances when compared to the Decipher or the CAPRA scores [40]. However, selecting patients at higher risk of bRFS or even metastatic-relapse does not necessarily mean that these patients would benefit more from aRT than from eSRT. Analyzing the performances of these advanced tools in the RAVES, RADICALS-RT and GETUG-AFU-17 trials would be interesting. A summary of the performances of these prognostic tools is available as Table 3. Before clinical implementation, these promising biological and diagnostic tools should be evaluated in clinical trials; the key point remaining the selection of patients that would possibly benefit from aRT on long-term endpoints.

## 7. Discussion

As presented, several key clinical issues were defined to summarize the abundance of literature on the aRT vs. (e)SRT debate. Data regarding aRT vs. SRT and aRT vs. eSRT were limited to published RCTs, while data regarding the impact of LNI and ADT were limited to RCTs or large and/or multicentric retrospective studies. This methodology has the advantage of a clear and concise report but with a risk of selection bias. However, the goal was mainly to discuss current literature data and offer practical insights on present and future perspectives in this clinical setting.

## 8. Conclusions

With a 5-years follow-up, adjuvant radiotherapy does not appear to significantly impact MFS and OS over eSRT in high-risk PCa patients. These aRT vs. eSRT RCTs confirmed the low value of bRFS as an intermediate surrogate of MFS and OS [41] and probably ended the time for the use of aRT at all. Duration of the follow-up is crucial when focusing on post-operative clinical endpoints where, with only observation, median bRFS ranges from 3.1 to 6.1 years and median OS is often immature due to the lack of follow-up. Clinicians should, thus, be careful not to completely bury aRT as some very selected patients might benefit from it. Delivered in patients with a very high-risk profile such as LNI or a combination of high-risk features (Decipher score > 0.6 or CAPRA-S score ≥ 8 or ≥2 risk factors such as invasion seminal vesicle invasion and positive surgical margins) but a long life expectancy and no post-operative dysfunction, aRT could be seen as an effective possibility to postpone ADT without compromising quality and quantity of life. Data regarding these very high-risk patients need further research. Selection of these patients is a challenge in a situation where clinical and pathological features are insufficient and where imaging modalities such as PET and MRI could be helpful. Translational research should be incorporated into the multidisciplinary management of these patients.

## Figures and Tables

**Table 1:** Key results from the adjuvant vs. salvage radiotherapy trials.

Trial	SWOG S8794 [4]			EORTC 22911 [1]			ARO 96-02/AUO AP 09/95 [5]		
Inclusion/Exclusion criteria	Inclusion criteria: pT3 pN0 or pNx Exclusion criteria: no exclusion criteria based on the post-operative PSA level			Inclusion criteria: pT2-pT3 + pN0 + at least one adverse feature capsular perforation positive surgical margins seminal vesicle invasion Exclusion criteria: no exclusion criteria based on the post-operative PSA level			Inclusion criteria: pT3 or pT4 pN0 Exclusion criteria: patients with a detectable post-operative PSA level were excluded		
Modality of radiotherapy	Prostate fossa: 60–64 Gy/30–32 fractions Pelvis: no ADT: no			Prostate fossa: 50 Gy/25 fractions Reduced volume: boost of 10 Gy/5 fractions Pelvis: no ADT: no			Prostate fossa: 60 Gy/30 fractions Pelvis: no ADT: no		
RT trigger	aRT: Randomization in the 16 weeks following surgery, start of RT in the 10 working days following randomization Observation: rising PSA			aRT: start of RT in the 16 weeks following surgery Observation: rising PSA			aRT: start of RT in the 6–12 weeks following surgery Observation: rising PSA		
Primary Endpoint	MFS			bRFS			PFS: biochemical recurrence, local or distant clinical recurrence or death of any cause		
Secondary Endpoints	bRFS OS PROs			cPFS OS PROs			bRFS OS PROs		
Population	Total: *n* = 425			Total: *n* = 1005			Total: *n* = 385		
	Observation: *n* = 211	aRT: *n* = 214	*p*	Observation: *n* = 503	aRT: *n* = 502	*p*	Observation: *n* = 159	aRT: *n* = 148	*p*
Pathology extent of disease									
Positive surgical margins only	-	-	-	15.7%	16.7%	0.73	-	-	-
Excapsular extension (ECE)	68% *	67% *	0.91	58.8%	57.4%	0.70	47%	51%	0.56
Seminal vesicle invasion (SVI)	11%	10%	0.86	25.4%	25.5%	0.97	17%	16%	0.93
Both ECE/positive margins and SVI	21%	23%	0.70	-	-	-	27%	27%	1.00
Invasion of surrounding organs	0.0%	0.0%	-	-	-	-	8%	3%	0.10
Pathology Gleason score									
2–6	46%	57%	0.03	WHO-Evaluated			36%	38%	0.81
7	38%	34%	0.45				54%	50%	0.56
8–10	16%	9.0%	0.04				10%	12%	0.71
Central pathology review	Incomplete: available for 73%			No			Incomplete: available for 85%		
Pre-RT PSA level									
Available data	186 (88.2%)	190 (88.8%)	0.97	502 (99.8%)	497 (99.0%)		-	-	-
<0.2 ng/mL	68%	65%	0.58	68.6%	70.3%		100%	100%	-
≥0.2 ng/mL	32%	35%		31.2%	28.7%		-	-	-
Percentage of performed RT	33.2%	100%?	<0.0001	30.8%	91.0%		?	(34 patients refused aRT)	-
Follow-up (median, years)	12.5 (IQR 11.1–14.0)	12.7 (IQR 11.4–15.1)	-	10.6 (IQR 8.4–12.5)			9.4 (IQR 7.2–10.8)	9.3 (IQR 7.3–10.7)	-
bRFS (median, years)	3.1	10.3	-	6.1	13.2	-	-	-	-
PFS (median, years)	-	-	-	-	-	-	4.9	Not reached	-
Proportion with 10-year MFS	61%	71%	0.04	71.3%	76.5%	0.07	-	-	-
Proportion with 10-year OS	66%	74%	0.09	80.7%	76.9%	0.16	-	-	-
Grade 2 or higher late genitourinary toxicity	9.5%	17.8%	0.02	13.5%	21.3%	0.003	0.0%	2.0% **	0.23 **
Grade 2 or higher late genitointestinal toxicity	0.0%	3.3%	0.02	1.9%	2.5%	0.47	0.0%	1.4%	0.42

* Patients with positives margins only (no ECE) could be included. ** Incontinence was not assessed. Abbreviations: wd: working days, RT: radiotherapy, aRT: adjuvant radiotherapy, SRT: salvage radiotherapy, ADT: androgen deprivation therapy, IQR: Inter-Quartile Range, bRFS: biochemical recurrence-free survival defined as the time from randomization to biochemical recurrence, MFS: metastasis recurrence-free survival, PFS: progression-free survival, cPFS: clinical progression-free survival, PROs: patient reported outcomes.

**Table 2:** Key results from the adjuvant vs. early salvage radiotherapy trials.

Trial		RADICALS-RT [8]			GETUG-AFU 17 [6]			RAVES [7]		
Inclusion/Exclusion criteria		Inclusion criteria: patients with at least one risk feature among: pT3 or pT4 Gleason score ≥ 7 Positive surgical margins Pre-operative PSA ≥ 10 ng/mL Exclusion criteria: patients with a detectable post-operative PSA level were excluded pN0/pNx was not an exclusion criteria			Inclusion criteria: pT3-pT4a pN0 or pNx positive surgical margins Exclusion criteria: patients with a detectable post-operative PSA level were excluded			Inclusion criteria: patients with at least one risk feature among: pT3a or pT3b positive surgical margins Exclusion criteria: patients with a detectable post-operative PSA level were excluded pN0/pNx was not an exclusion criteria		
Modality of radiotherapy		Prostate fossa: 66 Gy/33 fractions or 52.5 Gy/20 fractions Pelvis: at the physician’s discretion ADT: if participating in RADICALS-HD, random allocation to 0, 6 or 24 months of ADT			Prostate fossa: 66 Gy/33 fractions Pelvis: at the physician’s discretion ADT: 6 months for all patients			Prostate fossa: 64 Gy/32 fractions Pelvis: no ADT: no		
RT trigger		aRT: initiation within both 2 months of randomization and 26 weeks of radical prostatectomy Observation: initiation within 2 months of biochemical recurrence Start of RT could be delayed by up to 2 months in case of ADT			aRT: start of RT within 3–6 months of radical prostatectomy Observation: when BCR occurred			aRT: start of RT within 4 months of radical prostatectomy Observation: within 4 months of BCR		
Primary Endpoint		EFS			EFS			bRFS		
Secondary Endpoints		MFS OS Disease-specific survival PROs			MFS OS Acute and late toxicities Change in QOL			Time to initiation of ADT Time to local, regional and distant progression OS Acute and late toxicities		
Population		Total: *n* = 1396			Total: *n* = 424			Total: *n* = 333		
		Observation: *n* = 699	aRT: *n* = 697	*p*	Observation: *n* = 212	aRT: *n* = 212	*p*	Observation: *n* = 167	aRT: *n* = 166	*p*
Pathology extent of disease										
Positive surgical margin		63%	63%	-	-	-	-	68%	66%	0.79
Excapsular extension or positive margin (pT3a)		56%	58%	0.48	77%	77%	-	-	-	-
Seminal vesicle invasion (pT3b)		19%	18%	0.68	20%	21%	0.89	20%	19%	0.93
Invasion of surrounding organs (pT4)		1%	1%	-	2%	1%	0.65	-	-	-
Pathology Gleason score										
2–6		7%	7%	-	10%	10%	-	2%	4%	0.45
7		75%	77%	0.42	79%	82%	0.51	83%	81%	0.74
8–10		18%	16%	0.36	11%	8%	0.37	15%	15%	-
Lymph node involvement										
Involved		5%	4%	0.44	0%	0%	-	1%	0%	0.61
Not involved or unknown		95%	96%		100%	100%	-	100%	99%	
CAPRA-S risk group										
Low (0–2)		8%	8%	-	Not available			13%	13%	-
Intermediate (3–5)		55%	55%	-				60%	59%	0.94
High (≥6)		37%	37%	-				27%	29%	0.78
Central pathology review		No			No			Available but pathology reporting based on pathology results from local institution		
Percentage of performed RT		32.0%	93%	<0.0001	54%	97%	<0.0001	50%	95.8%	<0.0001
Follow-up (median, years)		4.9			6.2 (IQR 3.9–8.3)	6.5 (IQR 4.3–8.1)	-	6.1 (IQR 4.3–7.5)		-
Proportion with 5-year EFS		85%	88%	0.12	90%	92%	0.58	89%	86%	0.51
Proportion with 8-year EFS		-	-	-	-	-	-	79%	80%	0.93
MFS		Immature			Immature			-	-	-
Proportion with 8-year OS		Immature			Immature			97%	92%	0.08
Grade 2 or higher late genitourinary toxicity		-	-	-	7%	27%	<0.0001	54% *	70% *	0.002
Grade 2 or higher late genitointestinal toxicity		-	-	-	5%	8%	0.24	10% *	14% *	0.53
Late diarrhea	G1 or 2	8%	17%	<0.0001	7%	12%	-	-		
	G3	<1%	1%	-	0%	0%	-			
	G4	0%	<1%	-	0%	0%	-			
Late proctitis	G1 or 2	5%	13%	<0.0001	3%	8%	-	-		
	G3	<1%	1%	-	0%	1%	-			
	G4	0%	0%	-	0%	0%	-			
Late cystitis	G1 or 2	7%	13%	<0.0005	0%	0%	-	-		
	G3	1%	1%	-	0%	<1%	-			
	G4	0%	0%	-	0%	0%	-			
Late hematuria	G1 or 2	4%	12%	<0.0001	4%	12%	-	-		
	G3	<1%	4%	-	<1%	2%	-			
	G4	0%	0%	-	<1%	0%	-			
Late urethral stricture	G1 or 2	3%	6%	0.0025	6%	9%	-	-		
	G3	2%	4%	-	0%	<1%	-			
	G4	<1%	0%	-	0%	0%	-			

* For the RAVES trial, no differences were made between acute and late toxicities in the presented toxicity results. These rates are thus overestimated when compared to the GETUG-AFU-17 and the RADICALS-RT trials. Abbreviations: EFS: Event-Free survival (Event being defined as the occurrence of BCR, local or distant recurrence, death of any cause), BCR: Biochemical Recurrence, MFS: metastasis recurrence-free survival, PROs: patient reported outcomes, QOL: Quality of Life, IQR: Inter-Quartile Range.

**Table 3:** Performances of the novel biomarkers for the prediction of patients’ outcomes.

Prediction Tool	Endpoint	Result	Setting
Presalvage PSA level [27]	OS	HR 1.57, *p* = 0.004	Post-hoc analysis
Genomics-only [10]	MFS	HR 1.26, *p* < 0.001	Post-hoc analysis
	OS	HR 1.21, *p* < 0.001	
Radiomics-only [39]	bRFS	HR 5.5, *p* < 0.0001	External validation
Radiomics + Genomics [40]	bRFS	HR 1.6 *p* = 0.04	Multi-institutional validation

Abbreviations: PSA: Prostate-Specific Antigen, HR: Hazard Ratio, IC95%: 95% Confidence Interval, OS: Overall Survival, MFS: metastasis recurrence-free survival, bRFS: biochemical recurrence-free survival.
